# How much has the increase in atmospheric CO_2_ directly affected past soybean production?

**DOI:** 10.1038/srep04978

**Published:** 2014-05-15

**Authors:** Gen Sakurai, Toshichika Iizumi, Motoki Nishimori, Masayuki Yokozawa

**Affiliations:** 1 National Institute for Agro-Environmental Sciences, 3-1-3 Kannondai, Tsukuba, Ibaraki 305-8604, Japan; 2 Graduate School of Engineering, Shizuoka University, 3-5-1 Johoku Naka-ku, Hamamatsu 432-8561, Japan

## Abstract

Understanding the effects of climate change is vital for food security. Among the most important environmental impacts of climate change is the direct effect of increased atmospheric carbon dioxide concentration ([CO_2_]) on crop yields, known as the CO_2_ fertilization effect. Although several statistical studies have estimated past impacts of temperature and precipitation on crop yield at regional scales, the impact of past CO_2_ fertilization is not well known. We evaluated how soybean yields have been enhanced by historical atmospheric [CO_2_] increases in three major soybean-producing countries. The estimated average yields during 2002–2006 in the USA, Brazil, and China were 4.34%, 7.57%, and 5.10% larger, respectively, than the average yields estimated using the atmospheric [CO_2_] of 1980. Our results demonstrate the importance of considering atmospheric [CO_2_] increases in evaluations of the past effects of climate change on crop yields.

Mounting evidence that increasing atmospheric CO_2_ concentration ([CO_2_]) is changing global climate patterns^1^ is central to understanding the relationship between climate change and food security. To understand the effects of climate change on crop production, it is important to evaluate the effects of past climate change on crop yields. Among the various environmental factors that influence crop production, increases in atmospheric [CO_2_] is one of the most important because of its enhancement of photosynthetic rates^2^,^3^, a phenomenon known as the CO_2_ fertilization effect. In the last 30 years, the atmospheric [CO_2_] has increased by approximately 50 ppm^4^.

Previous statistical analyses of historical data on crop yields and climate conditions have revealed the important effects of temperature increases^5^,^6^,^7^,^8^,^9^. Few studies, however, have statistically delineated the independent effects of atmospheric [CO_2_] increases on past crop yields^10^. This is largely because the average temperatures during crop growing seasons have varied with space and time, which makes it possible to statistically distinguish between temperature effects and non-climatic effects (including the CO_2_ fertilization effect). It is difficult, however, to statistically isolate CO_2_ fertilization effects from the other effects because the average atmospheric [CO_2_] does not vary widely over space and time.

It may be possible to employ non-statistical approaches, using process-based crop models, to evaluate past CO_2_ fertilization effects^11^. However, simple simulation studies using process-based models may provide misleading results because of the uncertainty of the model parameters when estimating climatic effect in large scale^12^,^13^. Process-based models, if used to evaluate past effects of atmospheric [CO_2_] increases on large-scale crop yields, should consider the spatial variations of the parameters^12^ and the uncertainties associated with the down-regulation of the CO_2_ fertilization effect under field conditions^14^.

In the present study we applied a Bayesian statistical approach to the parameter estimation of a basic process-based model of soybean growth. In this approach, the spatial variability of the model parameters was considered by estimating the posterior distribution of the parameters from historical yield data using the Markov-chain Monte Carlo (MCMC)^15^ method for each grid cell.

Using this method, we estimated past CO_2_ fertilization effects in two steps. First, we updated the distributions of the model parameters using county-level historical data for crop yields from 1980 to 2006 in three major soybean-producing countries (the USA, Brazil, and China) with a spatial resolution of 1.125° × 1.125° (latitude × longitude). This Bayesian updating procedure enabled us to optimize our model for each grid cell by considering the uncertainties in the parameters. Second, we evaluated the effect of elevated atmospheric [CO_2_] on the soybean yields in the three countries from 1980 to 2006 by comparing the yields calculated by the models under historical climatic conditions with those under de-trended CO_2_ conditions in which the atmospheric [CO_2_] was fixed at the 1980 level (339 ppm).

## Results

We estimated the posterior distribution of some of the model parameters for each grid cell (total, 397) using historical yield data for soybeans; seven parameters were estimated for each cell. The parameters were related to the CO_2_ fertilization effect, corrections for technological differences among cells, drought stress, and cold stress (see Supplementary Information). Although the number of parameters estimated is somewhat large, several had prior distributions that limited their values to within realistic bounds (see Supplementary Information).

**Figure 1:**
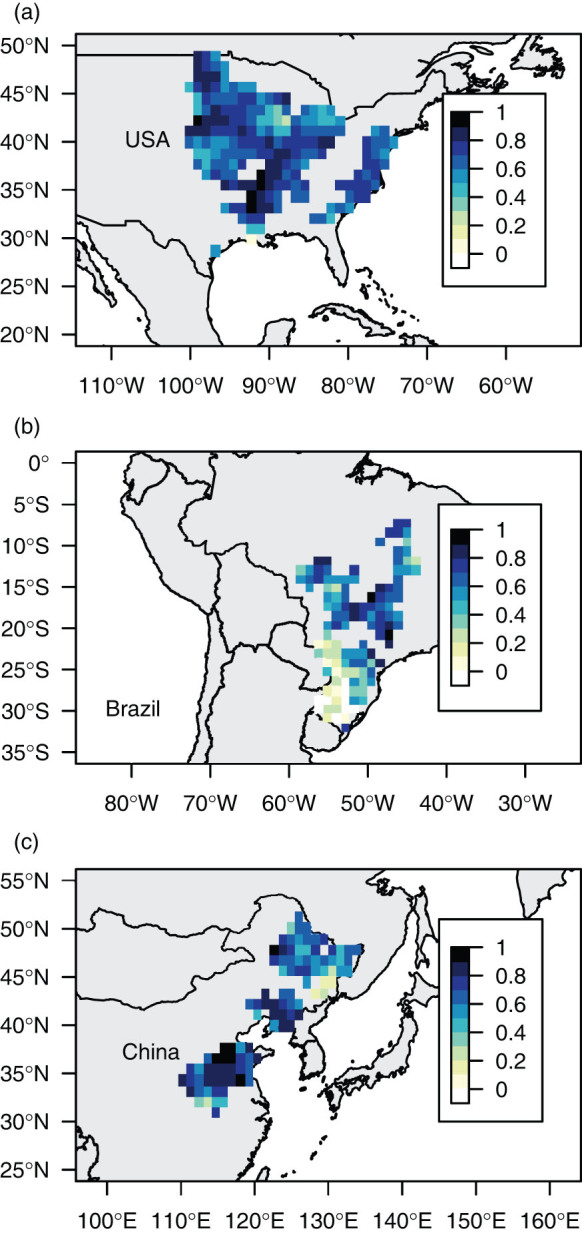
Correlation coefficients between estimated and observed crop yield. The values shown represent the correlation coefficients for the relationship between estimated and observed soybean crop yield from 1980 to 2006 (from 1990 to 2006 for Brazil) for each grid cell in (a) the USA, (b) Brazil, and (c) China. Estimated yield was calculated using a model in which the parameters were estimated for each cell using the Bayesian method. This figure was generated using R version 3.0.2 (http://www.R-project.org/).

By calibrating the parameters for each grid cell, we obtained spatially optimized models with a high capacity to estimate past observed yield. High correlations between historical and estimated yields were obtained for most areas of the three countries (Fig. 1), except in southern Brazil. The average correlation coefficient was 0.62 (SD, 0.22). The average RMSE was 0.30 t/ha (SD, 0.09).

**Figure 2:**
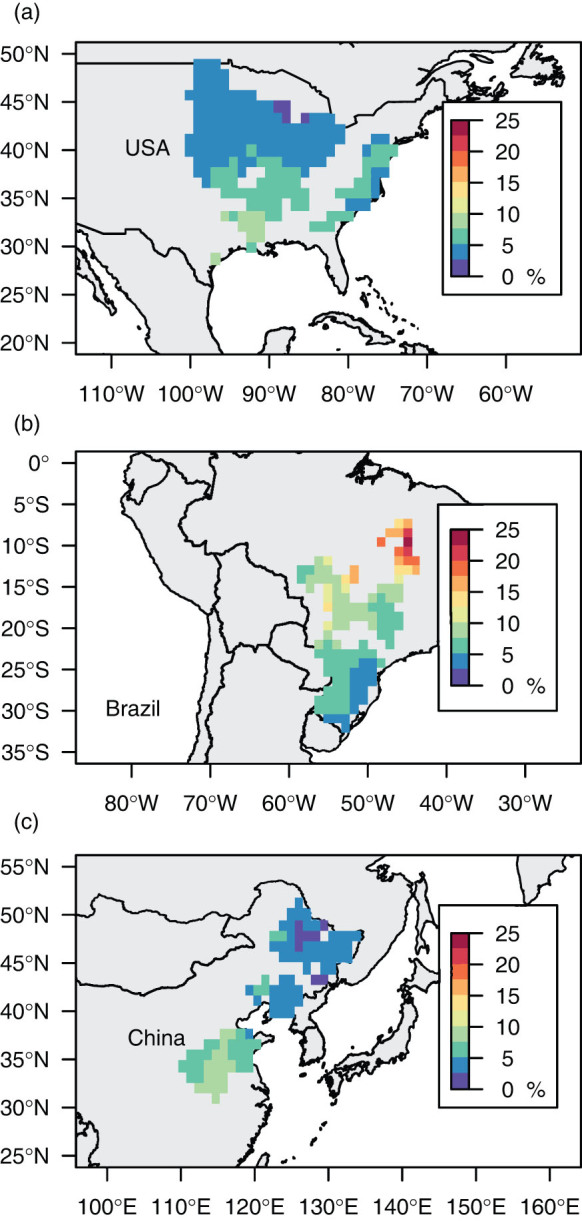
Spatial variability of the CO_2_ fertilization effect. The average impact of the CO_2_ fertilization effect (%) on soybean yield between 1980 and 2006 in each grid cell in (a) the USA, (b) Brazil, and (c) China. The values shown are averages for 2002–2006. The effect was estimated by comparing the estimated yield (t/ha) for 2002–2006 under historical climatic conditions with the estimated yield for the same five years using atmospheric CO_2_ conditions from 1980. This figure was generated using R version 3.0.2 (http://www.R-project.org/).

We next estimated the past CO_2_ fertilization effect by comparing the yields calculated by the models under historical climatic conditions with those under de-trended CO_2_ conditions in which the atmospheric [CO_2_] was fixed at the value for 1980. The CO_2_ fertilization effects during the past quarter century were estimated by comparing the estimated yield under historical climatic conditions for the last five years (2002–2006) with the estimated yield for these same years under atmospheric [CO_2_] conditions for 1980 (Fig. 2). We observed large spatial variation in the CO_2_ fertilization effect among cells. The average impact of the CO_2_ fertilization effect on soybean crop yield during the last quarter century was 0.11 t/ha in the USA, 0.19 t/ha in Brazil, and 0.10 t/ha in China. These values correspond to respective increases in average yield attributable to increases in atmospheric [CO_2_] of 4.33%, 7.58%, and 5.05%.

**Figure 3:**
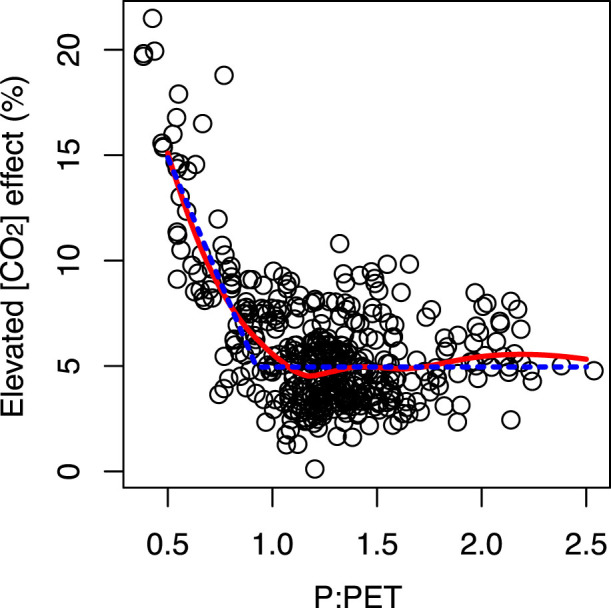
Relationship between the average CO_2_ fertilization effect on crop yield between 1980 and 2006 and the ratio of precipitation to potential evapotranspiration (P:PET) during the growing season in each grid cell. PET was estimated according to Thornthwaite (1948)^30^. The red line was generated using local regression. The blue line represents the model selected by AIC model selection analysis; it shows an inflection point around P:PET = 0.92.

The magnitude of the CO_2_ fertilization effect was positively correlated with the average solar radiation during the growing period (Kendall's rank correlation test; τ = 0.18, *P* < 0.001); negatively correlated with the maximum leaf area index (LAI) for the cells (τ = −0.59, *P* < 0.001); and negatively correlated with the ratio of precipitation to potential evapotranspiration (P:PET) during the growing season (τ = −0.23, *P* < 0.001) (Fig. 3).

To estimate the inflection point where the CO_2_ fertilization effect starts to increase with increasing P:PET, we estimated the parameters for three models: a simple linear model (model 1), a model with two intersecting straight lines, both of which had a slope (model 2), and a model with two intersecting straight lines, one of which had a slope of zero (model 3). Model 3 was selected at the best model on the basis of the Akaike information criterion (AIC) values (Fig. 3). The estimated inflection point was at P:PET = 0.92.

## Discussion

We found large increases in soybean yields over the past 27 years due to the CO_2_ fertilization effect. The average effect of elevated atmospheric [CO_2_] on soybean yield during the last quarter century (*i.e*., the difference between the average yield for 2002–2006 estimated using historical CO_2_ levels and the average yield for 2002–2006 estimated using 1980 CO_2_ levels) was 0.13 t/ha. This shows that soybean yields during 2002–2006 have increased by 5.84% on average as a result of corresponding increases in atmospheric [CO_2_] from 1980.

The effect was slightly higher than that suggested by the results of previous free-air CO_2_ enrichment (FACE) studies, in which an increase in atmospheric [CO_2_] of approximately 180 ppm gave an average increase of approximately 14%^16^. If there is a linear relationship between atmospheric CO_2_ levels and the CO_2_ fertilization effect, then the expected CO_2_ fertilization effect from 1980 (atmospheric [CO_2_], 338 ppm) to 2006 (377 ppm) would be approximately 3%. However, the relationship between atmospheric [CO_2_] and the CO_2_ fertilization effect is not linear^17^. Considering the response curve of photosynthesis^17^ to [CO_2_], our results for this relationship are not inconsistent with previous reports. In addition, McGrath and Lobell (2011)^10^ suggested that the CO_2_ fertilization effect can more than double under conditions of water stress relative to that in the absence of water stress; this would also increase the average of the historical CO_2_ fertilization effect at a global scale above that expected simply from the FACE experiment (3%).

The spatial variation of the CO_2_ fertilization effect was associated with certain environmental conditions and specific crop characteristics. The positive correlation between the magnitude of the CO_2_ fertilization effect and the average solar radiation during the growing period might be explained by the fact that crops cannot receive the maximum benefits of increased [CO_2_] in an area with limited solar radiation. The negative correlation with the maximum LAI of the grid cells might be explained by differences in growth associated with LAI. When leaf area is small, increases in the daily rate of photosynthesis result in exponential increases in biomass; however, as leaf area increases, the proportion of photosynthetically active radiation (PAR) absorbed by leaves per unit area gradually decreases^18^; for example, although an increase in LAI from 6.0 to 7.0 (an increase of about 17%) would result in an increase in absorbed PAR of only 2.1%, a 17% increase in LAI from 4.0 to 4.7 would result in an increase of 4.4%, assuming an extinction coefficient of 0.5 and the Beer's law approximation. Finally, the magnitude of the CO_2_ fertilization effect was negatively correlated with the P:PET ratio during the growing season. Elevated atmospheric [CO_2_] decreases stomatal conductance, which can result in increased soil moisture. This could explain the greater CO_2_ fertilization effect in dry areas in our study^10^.

**Figure 4:**
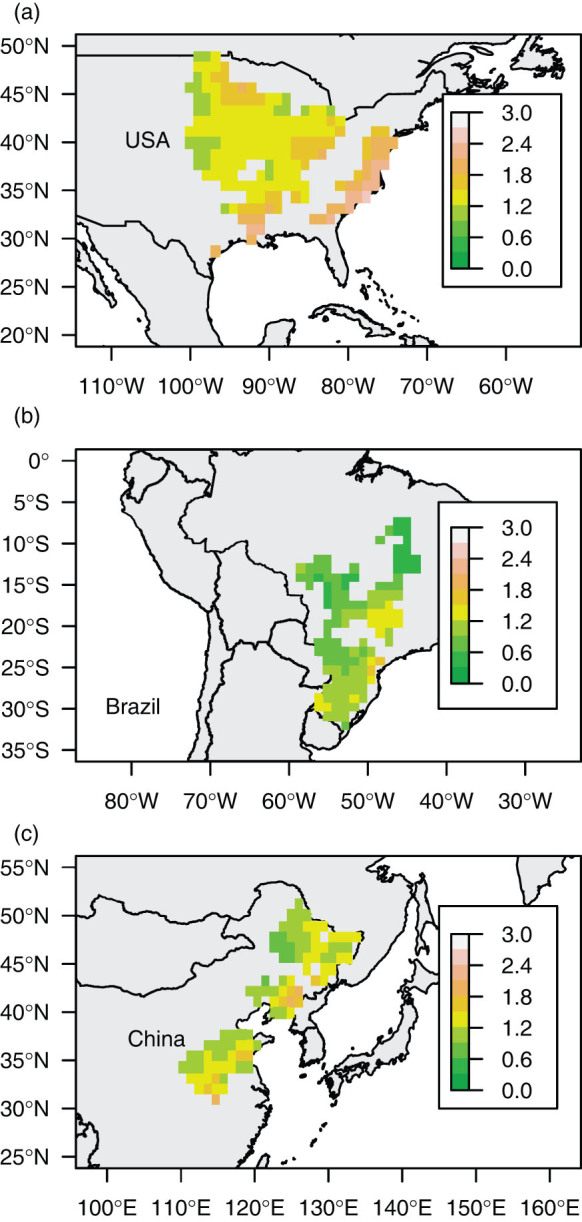
Spatial variability of P:PET. The value of P:PET averaged for 1980–2006 is shown for each grid cell in (a) the USA, (b) Brazil, and (c) China. This figure was generated using R v. 3.0.2 software (http://www.R-project.org/).

From the effects of these mechanisms, we would expect the CO_2_ fertilization effect to be higher in the northern part of Brazil and in the southern parts of the USA and China than in the other areas in this study, because P:PET values were lower in northern Brazil (Fig. 4) and the maximum LAI values of grid cells were lower in the southern USA and China.

One possible criticism of this study is that our model includes only relatively robust processes; for example, it incorporates a “big leaf” model and Farquhar's model. We excluded several processes from the model, such as nitrogen dynamics, allocation of photosynthetic products, insect damage, and cold-weather damage. Moreover, although soybean is a forage crop that establishes a symbiotic relationship with a nitrogen-fixing bacterium, nitrogen supply can inhibit the acclimatization of photosynthesis to elevated [CO_2_], even for legumes^19^. However, there are no historical forcing data that cover the entire world during the entire study period that could be used to simulate such detailed processes, and there is no guarantee that modelling of such detailed processes would be applicable worldwide. Moreover, it would be difficult to determine more parameter values using only the historical yield data. We believe that the degree of simplicity of the model in this study is the good compromise between complexity and robustness for calculating a global-level yield response to climate change.

In this study, the distributions of the several parameters of the model were estimated using historical yield data for each grid cell. This procedure enabled us to optimize our model for each cell. However, in several cells, particularly in southern Brazil, there were poor correlations between historical and modelled yields. One possible reason is that the historical yields in Brazil may have responded to factors not considered in our model.

In this context, another possible criticism of this study is that we used only one model to estimate the CO_2_ fertilization effect, and it might therefore be useful to apply the same procedure to other crop models to estimate CO_2_ fertilization effects on past crop yields. Because many models adopt Farquhar's model^17^ for the photosynthesis–CO_2_ response curve, it is unlikely that estimates of the CO_2_ fertilization effect based on other models would differ substantially from those reported in this study. Even so, it would be worth confirming our results by applying our methodology to other models.

## Methods

### Model

We used robust models for the estimation of soybean growth. The photosynthetic carbon assimilation was calculated by the enzyme kinetics model developed by Farquhar et al^17^. For simplicity, we assumed that soybean production is not limited by levels of nitrogen and other nutrients^20^. We used a “big-leaf” model^21^ in which each plant is treated as a single big leaf. Soil water content and drought stress were calculated by the Soil & Water Assessment Tool^22^. The model description is provided in the Supplementary Information.

### Data

The data from a FACE experiment were obtained from Morgan et al^23^. We used their aboveground biomass and leaf biomass data for the Bayesian estimation of the parameters. The historical yield data for each cell were obtained from the United States Department of Agriculture (USDA) (http://www.usda.gov/wps/portal/usda/), the Brazilian Institute of Geography and Statistics (http://www.ibge.gov.br/home/), and the National Bureau of Statistics of China. These three countries account for approximately 68% of the world soybean production^24^. Although the yield data for China covered only four provinces, these provinces account for 51% of the net soybean production in China^25^. For climate data, we used the Japanese re-analysis dataset^26^, which covers regions across the globe at a spatial resolution of 1.125° × 1.125° (latitude × longitude). We estimated the crop yields for each grid cell by averaging the yield data for the counties or municipalities included in the cell. We used only grid cells that have more than 1% of the soybean harvest areas^27^ in the cells and including more than 14 years of yield data with no more than 3 consecutive years of missing data.

### Parameter estimation using FACE data

We estimated parameter values from FACE data using the Markov-chain Monte Carlo (MCMC) method with simulated tempering^28^. The estimated parameters are listed in Supplementary Tables S1 and S2 online. The number of MCMC steps was set to 200 000, with 20 tempering temperatures for each prospective model.

### Parameter estimation using county-level data

The parameters were updated with the historical yield data for the three countries included in this study using the DiffeRential Evolution Adaptive Metropolis (DREAM) algorithm^29^. The calculation was performed independently for each grid cell. We assumed a linear trend for the effects of improvements in agricultural management on the soybean yield, and estimated the slope of the linear trend for each grid cell together with the other model parameters (see Supplementary Information). The estimated linear trend for the effects of agricultural improvements included all technological progress (but no climatic factors), such as changes in fertilizer input, the repertoire of pesticides, soil improvement techniques, and crop cultivars. The estimated parameters are listed in Supplementary Table S3 online. The number of MCMC steps was set to 50 000 with 10 chains for each cell. To estimate the posterior distributions of the parameters using the historical yield data from the grid cell, we assumed a multivariate normal distribution for the error distribution of soybean yield. We set the non-diagonal element of the variance–covariance matrix of the error distribution to zero; *i.e*., we assumed no correlation among the errors.

### Estimation of CO_2_ fertilization effect

The past CO_2_ fertilization effect was estimated by comparing the yields estimated under historical weather and atmospheric [CO_2_] conditions with those estimated under conditions in which the atmospheric [CO_2_] was fixed at the 1980 level. The effects of past temperature, precipitation, and solar radiation trends were estimated in a similar manner. The trends of temperature, precipitation, and solar radiation were estimated using simple linear regression analysis of data pertaining to the crop-growing season.

## Author Contributions

G.S. and M.Y. conceived the study; T.I. collected the data; G.S. analysed the data and drafted the paper; and G.S., I.T., M.N. and M.Y. contributed to the writing of the manuscript.

## Supplementary Material

Supplementary Information Supplementary Information
