# Supplementary material for: How much has the increase in atmospheric CO2 directly affected past soybean production?
Source: Sci Rep. 2014 May 15;4:4978. doi: 10.1038/srep04978 (PMC4021318; doi:10.1038/srep04978)
Supplement: Supplementary Information [file srep04978-s1.pdf]

Supplementary Information

How much has the increase in atmospheric CO_2 directly affected past crop production?

Gen Sakurai[1], Toshichika Iizumi[1], Motoki Nishimori[1], and Masayuki Yokozawa[2]*

[1] National Institute for Agro-Environmental Sciences, 3-1-3 Kannondai, Tsukuba, Ibaraki 305-8604, Japan

[2] Graduate School of Engineering, Shizuoka University, 3-5-1 Johoku Naka-ku, Hamamatsu 432-8561, Japan

*Corresponding author: Masayuki Yokozawa
E-mail address: yokozawa@sys.eng.shizuoka.ac.jp
Phone & Fax: +81-53-4781218

Supplementary Equations and Methods

Model Description

1. Summary of the model

The model was developed for this study to simulate soybean growth. The purpose of developing this process-based model was to evaluate the effect of increased atmospheric CO_2 concentrations on crop yields due to the influence of atmospheric CO_2 and other environmental factors on enzyme kinetics. In this model, certain model parameters were estimated using Bayesian statistics with a Markov-Chain Monte Carlo (MCMC) method based on field observation data. This process allows for the model parameters to be calibrated to fit any region or scale[1]. The model repeatedly performs hundreds of thousands of calculation steps in the MCMC process. Therefore, although the model must be sufficiently complex to describe the response of soybean production to increased atmospheric CO_2, it should be relatively simple and include the fewest parameters possible to shorten the calculation time for each MCMC step. To this end, we simplified the model in the following manner:

i. Although the simulation of photosynthetic carbon assimilation was based on the enzyme kinetics model originally developed by Farquhar *et al.*[2], we used an analytical solution for the photosynthesis and stomatal conductance model[3] to avoid the need for extensive iteration during the calculation of the photosynthetic carbon assimilation rate[4].

ii. We assumed that soybean production was not limited by the availability of nitrogen or other nutrients given that soybean crops are generally well fertilized[5].

iii. We used a "big-leaf" model[6] to avoid having to determine the leaf micrometeorological environment.

2. Input data

For input data, this model used the planting date in each year, average daily temperature, maximum and minimum daily temperatures, total daily downward solar radiation at the surface, and daily precipitation. We used a two-layer sub-model of the Soil & Water Assessment Tool (SWAT) model[7] but did not include lateral transport to calculate the soil water balance. Therefore, the model also used the input data required by the SWAT model.

3. Crop growth

The crop growth component of the model was partly based on existing models, such as SiB2[8,9]. However, for the purposes of this study, all aspects of the crop development process were simplified as much as possible, with the exception of the photosynthetic process.

3.1. Growing period

The time of seedling emergence after the planting date was determined as follows:

$$DOY_{emerge} = DOY_{plant} + l_{emerge}, \quad (1)$$

where DOY_{emerge} is the day of the year of soybean seedling emergence, DOY_{plant} is the day of the year of soybean planting, and l_{emerge} is the average duration (in days) of the period between planting and seedling emergence. To simplify the model structure, we described the average period between planting and emergence using l_{emerge}. This simplification is acceptable when the same type of agricultural management is used during consecutive years[10].

The period of time from emergence to maturity is determined by the total number of heat units[7]:

$$HU_{td}=\min(0, TM_{td}-tm_{base}), \quad (2)$$

$$THU_{td} = \sum_{i=DOY_{emerge}}^{td} HU_i, \quad (3)$$

where HU_{td} is the heat unit (°C) on a given day (td), TM_{td} is the average temperature (°C) on a given day (td), tm_{base} is the base temperature (°C), and THU_{td} is the total number of heat units (°C days) accumulated during the period up to the given day (td). The crop is mature when THU_{td} is equal to thu_{total}, at which point it is harvested. Using the biomass values obtained at the time of crop maturity, yield is calculated as follows:

$$Yield=hi \times BIO_{above(maturity)}, \quad (4)$$

where Yield is the crop yield (kg ha^{-1}), hi is the harvest index, and $BIO_{above(maturity)}$ (kg ha^{-1}) is the aboveground biomass at the time of crop maturity. hi changes according to the atmospheric CO_2 concentration[11] as follows:

$$hi=hi_{base}\left[1+coef_{hi}\left(\frac{C_a}{c_{base}}-1\right)\right], \quad (5)$$

where hi_{base} is the harvest index at the standard CO_2 concentration, c_{base}; C_a is the atmospheric CO_2 concentration; and $coef_{hi}$ is the parameter adjusting the effect of the CO_2 concentration on hi. $coef_{hi}$ is the degree of reduction of hi when the CO_2 concentration is twice the base concentration (for example, a value of –0.1 indicates a 10% reduction in *hi*). The value of $coef_{hi}$ was determined according to Ainsworth *et al.*[11].

3.2. Biomass production

In the biomass production component of the model, we used an analytical solution for the photosynthesis submodel[3]. In addition, an abbreviated process was used for the daily allocation of photosynthate to plant organs. As a result, we were able to reduce the number of iterations required to simulate the photosynthetic process, and we reduced the number of parameters to be estimated.

3.2.1. Initial biomass

The initial biomass (kg ha^{-1}) on the day of seedling emergence is specified by the parameter bio_{init}.

3.2.2. Leaf area index

Leaf area index (LAI) on a given day is calculated based on leaf biomass:

$$LAI_{td} = BIO_{leaf(td)} \times sla, \quad (6)$$

where LAI_{td} is the LAI (ha ha^{-1}) on a given day (td), $BIO_{leaf(td)}$ is the leaf biomass for that day (kg ha^{-1}), and sla is the specific leaf area (ha kg^{-1}). sla changes according to the CO_2 concentration as follows:

$$sla = sla_{base}\left[1 + coef_{sla}\left(\frac{C_a}{c_{base}} - 1\right)\right], \quad (7)$$

where sla_{base} is the specific leaf area at the standard CO_2 concentration, c_{base}; C_a is the atmospheric CO_2 concentration; and $coef_{sla}$ is the parameter adjusting the effect of the CO_2 concentration on sla. The value of $coef_{sla}$ was determined according to Ainsworth *et al.*[11].

The ratio of leaf biomass to aboveground biomass, hereafter referred to as the 'leaf ratio,' is linearly correlated with the ratio of THU_{td} to thu_{total} ($Fthu_{td}$). The relationship was assumed to be stable among years, varieties, and treatments (see Section 8). Therefore, the following equation can be derived:

$$Rate_{leaf(td)} = l - l\left(\frac{THU_{td}}{thu_{total}}\right), \quad (8)$$

where $Rate_{leaf\,(td)}$ is the ratio of leaf biomass to aboveground biomass on a given day (td) and l is a parameter that defines the intercept and the gradient. Using this simple leaf ratio equation reduces uncertainty due to the determination of the allocation of photosynthesis products to each organ and shortens calculation times.

We used the same ratio of root biomass to total biomass that is assumed in the SWAT model[7]. The ratio varies from 0.40 at emergence to 0.20 at maturity[12], and the daily ratio on a given day td, $Rate_{root(td)}$, is calculated as:

$$Rate_{root(td)} = 0.40 - 0.20\left(\frac{THU_{td}}{thu_{total}}\right). \quad (9)$$

Using the biomass values on a given day (BIO_{td}), $Rate_{root(td)}$, and the ratio of leaf biomass to crop biomass for that given day ($Rate_{leaf(td)}$), root biomass, aboveground biomass, and leaf biomass can be calculated.

3.2.3. Time course of solar radiation and air temperature

If global radiation data are available at a daily resolution, the instantaneous global radiation at a particular time (*t*) of day can be estimated from the daily global radiation on a given day (td), H_{td} (MJ m^{-2} day^{-1}), according to the method described by Goudriaan and van Laar[13].

$$H_{t,td} = H_{td} \frac{\sin\beta\left[1 + 0.033\cos\left(\frac{2\pi\left(\text{DOY-10}\right)}{365}\right)\right]}{D}, \quad (10)$$

where $H_{t,td}$ is the instantaneous global radiation (MJ m^{-2} s^{-1}) at time *t* and DOY is the day of the year (beginning January 1st). The values of sinβ and *D* are given by the following equations:

$$\sin\beta = A + B \times \cos\left[\frac{2\pi\left(\text{STime}_t - 12\right)}{24}\right], \quad (11)$$

$$D = 3600\left[D_{la} \times A + B\left(\frac{24}{\pi}\right)\sqrt{1 - \left(\frac{A}{B}\right)^2}\right], \quad (12)$$

where STime_t is the time of day (solar time) at time *t* and *A* and *B* are given by the following equations:

$$A = \sin\left(\frac{\pi \times \text{LAT}}{180}\right)\sin\Delta, \quad (13)$$

$$B = \cos\left(\frac{\pi \times \text{LAT}}{180}\right)\sin\Delta, \quad (14)$$

where LAT is the latitude (rad) and Δ is defined as

$$\Delta = -\arcsin\left\{\sin\left(\frac{23.45\pi}{180}\right)\cos\left[\frac{2\pi\left(\text{DOY} + 10\right)}{365}\right]\right\}. \quad (15)$$

D_{la} (the duration from sunrise to sunset) (hours) is given by the following equation:

$$D_{la} = 12\left\{1 + \left(\frac{2}{\pi}\right) \times \arcsin\left(\frac{A}{B}\right)\right\}. \quad (16)$$

A simpler approach was used to derive the diurnal temperature course (°C) based on the daily maximum and minimum temperatures[14]:

$$\mathrm{TM}_{t,\mathrm{dt}} = 0.5\left\{\left(\mathrm{TX}_{\mathrm{td}} + \mathrm{TN}_{\mathrm{td}}\right) + \left(\mathrm{TX}_{\mathrm{td}} - \mathrm{TN}_{\mathrm{td}}\right)\cos\left[\frac{\pi\left(\mathrm{ITime}_t - 8\right)}{12}\right]\right\}, \quad (17)$$

where $\mathrm{TM}_{t,\mathrm{dt}}$ is the air temperature (°C) at time t on a given day td, $\mathrm{TX}_{\mathrm{td}}$ is the maximum daily temperature (°C), $\mathrm{TN}_{\mathrm{td}}$ is the minimum daily temperature (°C), and ITime_t is the time of day (hours, starting with 1 for 07:00). The photosynthetic rate is estimated hourly and summed as follows:

$$\mathrm{As}_{\mathrm{td}} = \sum_{\mathrm{ITime}_t=1}^{24} 3600\mathrm{As}_t, \quad (18)$$

where As_t is the instantaneous photosynthetic rate (μmol m^{-2} s^{-1}) at time t, estimated every hour, and $\mathrm{As}_{\mathrm{td}}$ is the total photosynthetic rate (μmol m^{-2} day^{-1}) for a given day (td). The potential mass of photosynthetic products on a given day is calculated as follows:

$$\Delta\mathrm{PBio}_{\mathrm{td}} = \frac{0.00014\mathrm{As}_{\mathrm{td}}}{c_{\mathrm{rate}}}, \quad (19)$$

where $\Delta\mathrm{PBio}_{\mathrm{td}}$ is the mass of photosynthetic products (kg ha^{-1} day^{-1}) on a given day and c_{rate} is the amount of carbon (kg kg^{-1}) in the crop biomass. The amount of photosynthetic products used for biomass growth on a given day is calculated as follows:

$$\Delta\mathrm{Bio}_{\mathrm{td}} = (\mathrm{bio}_{\mathrm{coef}(C\mathrm{a})} \times \Delta\mathrm{PBio}_{\mathrm{td}} - \mathrm{RM}_{\mathrm{root(td)}} - \mathrm{RM}_{\mathrm{stem,pod(td)}} - \mathrm{RG}_{\mathrm{td}}), \quad (20)$$

where $\Delta\mathrm{Bio}_{\mathrm{td}}$ is the actual biomass growth (kg ha^{-1} day^{-1}), $\mathrm{RM}_{\mathrm{root(td)}}$ is the maintenance respiration of roots on a given day (kg ha^{-1} day^{-1}), $\mathrm{RM}_{\mathrm{stem,pod(td)}}$ is the maintenance respiration rate of plant stems and pods (kg ha^{-1} day^{-1}), $\mathrm{RG}_{\mathrm{td}}$ is the growth respiration rate on a given day (kg ha^{-1} day^{-1}), and $\mathrm{bio}_{\mathrm{coef}(C\mathrm{a})}$ is the biomass growth compensation coefficient when [CO_2] is C_a (see section 3.4).

3.2.4. Photosynthesis

The amount of photosynthetically active radiation, $\mathrm{PAR}_{t,\mathrm{td}}$ (MJ m^{-2} s^{-1}), intercepted by the leaf at a time t on a given day td is calculated using Beer's law[15]:

$$\mathrm{PAR}_{t,\mathrm{td}} = 0.5 \times H_{t,\mathrm{td}}\left[1 - \exp\left(-k \times \mathrm{LAI}_{\mathrm{td}}\right)\right], \quad (21)$$

where $H_{t,\mathrm{td}}$ is the instantaneous global radiation (MJ m^{-2} s^{-1}) at time t on a given day td, k is the light extinction coefficient within the soybean canopy, and $\mathrm{LAI}_{\mathrm{td}}$ is the leaf area index on a day td. The potential rate of electron transport at that time, $J_{t,\mathrm{td}}$ (μmol m^{-2} s^{-1}), was calculated according to following equation[16]:

$$\theta J_{t,\mathrm{td}} - \left(\alpha Q + J_{\max(t,\mathrm{td})}\right) J_{t,\mathrm{td}} + \alpha Q J_{t,\mathrm{td}} = 0\,, \quad (22)$$

where $J_{\max}$ is the potential rate of electron transport at time t on a given day td; Q is the photosynthetically active photon flux, which is calculated using the conversion factor for solar radiation, cf, at 550 nm (green) and $PAR_{t,\mathrm{td}}$; θ is the curvature of the light response curve; and α is the quantum yield of electron transport (mol electrons mol^{-1})[16].

We used the model described by Baldocchi[3] to calculate the photosynthetic rate as summarized below. The leaf photosynthetic rate (As) (μmol m^{-2} s^{-1}) is a function of the carboxylation (V_c), oxygenation (V_o), and dark respiration (R_d) rates:

$$\mathrm{As} = V_c - 0.5V_o - R_d\,. \quad (23)$$

The response of photosynthesis to absorbed PAR under PAR-limited conditions is calculated as

$$V_c - 0.5V_o = \frac{J_{t,\mathrm{td}}\left(C_i - \Gamma\right)}{4C_i - 8\Gamma}\,, \quad (24)$$

where $J_{t,\mathrm{td}}$ is the potential rate of electron transport (μmol m^{-2} s^{-1}) at time t on a given day td, C_i is the internal [CO_2] (mol mol^{-1}), and Γ is the CO_2 compensation point (mol mol^{-1}) in the absence of dark respiration. The Rubisco-limited rate of photosynthesis is calculated as

$$V_c - 0.5V_o = \frac{V_{\mathrm{cmax}(t,\mathrm{td})}\left(C_i - \Gamma\right)}{C_i + K_c\left(1 + \frac{[O_2]}{K_o}\right)}\,, \quad (25)$$

where $V_{\mathrm{cmax}(t,\mathrm{td})}$ is the maximum carboxylation rate of Rubisco (μmol m^{-2} s^{-1}) at time t on a given day td; K_c and K_o are the Michaelis-Menten coefficients (mol mol^{-1}) for CO_2 and O_2, respectively; and [O_2] is the O_2 concentration (mol mol^{-1}). C_i is influenced by the value of As as follows:

$$C_i = C_s - \frac{\mathrm{As}}{G_s}\,, \quad (26)$$

$$C_s = C_a - \frac{\mathrm{As}}{G_a}\,, \quad (27)$$

where C_s is the leaf-surface atmospheric CO_2 (mol mol^{-1}), G_s is the stomatal CO_2 conductance (μmol m^{-2} s^{-1}), C_a is the atmospheric CO_2 (mol mol^{-1}), and G_a is the CO_2 conductance across the laminar boundary layer of the leaf (μmol m^{-2} s^{-1}). We cannot obtain an analytical solution for As using only these equations. However, by assuming the following relationship for G_s[17], we can obtain an analytical solution for As[3]:

$$G_s = \frac{m \times \mathrm{As} \times \mathrm{RH}}{C_s} + b', \quad (28)$$

where m is a dimensionless slope coefficient, RH is the relative humidity, and b' is the zero intercept when As ≤ 0. In the photosynthesis model, the values of the parameters were the same as those of Sellers *et al.*[9,10] except for the parameters listed in Table S1.

3.2.5. Temperature stress

The influence of temperature on the maximum carboxylation rate of Rubisco is given as follows:

$$C_{\mathrm{vcmax}(t,\mathrm{td})} = \exp\left[\frac{\mathrm{adj_E} E_{\mathrm{vcmax}}\left(\mathrm{TM}_{t,\mathrm{td}} - 25\right)}{298R \cdot \left(\mathrm{TM}_{t,\mathrm{td}} + 273\right)}\right], \text{ and} \quad (29)$$

$$C_{\mathrm{jmax}(t,\mathrm{td})} = \exp\left[\frac{\mathrm{adj_E} E_{\mathrm{jmax}}\left(\mathrm{TM}_{t,\mathrm{td}} - 25\right)}{298R \cdot \left(\mathrm{TM}_{t,\mathrm{td}} + 273\right)}\right] \frac{1 + \exp\left(\frac{298S_{\mathrm{jmax}} - H_{\mathrm{jmax}}}{298R}\right)}{1 + \exp\left[\frac{\left(\mathrm{TM}_{t,\mathrm{td}} + 273\right)S_{\mathrm{jmax}} - H_{\mathrm{jmax}}}{\left(\mathrm{TM}_{t,\mathrm{td}} + 273\right)R}\right]}, \quad (30)$$

where $C_{\mathrm{vcmax}(t,\mathrm{td})}$ and $C_{\mathrm{jmax}(t,\mathrm{td})}$ represent the effect of temperature on the maximum carboxylation rate of Rubisco and the potential rate of electron transport, respectively; $\mathrm{TM}_{t,\mathrm{td}}$ is the air temperature (°C) at time t on day td; $\mathrm{adj_E}$ is the adjustment parameter changing both E_{vcmax} and E_{jmax} that is introduced to reduce the number of parameters that are relevant to temperature sensitivity in this study; E_{vcmax}, E_{jmax}, S_{jmax}, and H_{jmax} are parameters that describe the shape of the curve[16]; and R is the universal gas constant (8.314 J mol^{-1} K^{-1}).

The influence of temperature on the dark respiration of leaves is given as

$$C_{\mathrm{dark}(t,\mathrm{td})} = q^{\left(\frac{\mathrm{TM}_{t,\mathrm{td}} - 25}{10}\right)} \left\{1 + \exp\left[s_3\left(\mathrm{TM}_{t,\mathrm{td}} - s_4\right)\right]\right\}, \quad (31)$$

where $C_{\mathrm{dark}(t,\mathrm{td})}$ represents the effect of temperature on dark respiration at time t on day td and q, s_1, and s_2 are parameters that describe the shape of the curve[8,9].

The maximum carboxylation rate of Rubisco, the potential rate of electron transport, and the dark respiration rate are modified by temperature effects that can be calculated using equations 29-31[8,9]:

$$V_{\mathrm{cmax}(t,\mathrm{td})} = C_{\mathrm{vcmax}(t,\mathrm{td})} \times W_{\mathrm{stress(td)}} \times v_{\mathrm{cmax}}, \quad (32)$$

$$J_{\mathrm{max}(t,\mathrm{td})} = C_{\mathrm{jmax}(t,\mathrm{td})} \times W_{\mathrm{stress(td)}} \times j_{\mathrm{max}}, \quad (33)$$

where $V_{\mathrm{cmax}(t,\mathrm{td})}$ is the maximum carboxylation rate of Rubisco and $J_{\mathrm{max}(t,\,\mathrm{td})}$ is the potential rate of electron transport, which is substituted into equation 24 and 25; $W_{\mathrm{stress(td)}}$ is the water stress,

which ranges from 0 for complete drought to 1 for no stress, on a given day (calculated using the SWAT submodel (see section 3.3)); and v_{cmax} and j_{max} is the potential maximum carboxylation rate and the potential rate of electron transport, respectively. The dark respiration rate is calculated as follows:

$$R_{\mathrm{d}(t,\mathrm{td})} = \mathrm{rd} \times C_{\mathrm{dark}(t,\mathrm{td})} \times v_{\mathrm{cmax}}, \quad (34)$$

where $R_{\mathrm{d}(t,\mathrm{td})}$ is the dark respiration rate (μmol m^{-2} s^{-1}), which is substituted into equation 23, and rd is the leaf respiration factor[4,8,9].

3.2.6. Maintenance and growth respiration

The maintenance respiration of stems and roots on a given day td is calculated as follows:

$$RM_{\mathrm{root(td)}} = \sum_{j=1}^{\mathrm{nsol}} rm \times C_{\mathrm{root},j(\mathrm{td})} \times BIO_{\mathrm{root},j(\mathrm{td})}, \quad (35)$$

$$\mathrm{RM}_{\mathrm{stem,pod(td)}} = \mathrm{rm} \times C_{\mathrm{stem,pod(td)}} \times \mathrm{BIO}_{\mathrm{stem,pod(td)}}, \quad (36)$$

where $\mathrm{RM}_{\mathrm{root(td)}}$ is the maintenance respiration of roots on a given day (kg ha^{-1} day^{-1}), $\mathrm{RM}_{\mathrm{stem,pod(td)}}$ is the maintenance respiration of stems and pods on that day (kg ha^{-1} day^{-1}), rm is the maintenance respiration rate (kg kg^{-1} day^{-1}), $C_{\mathrm{root},j(\mathrm{td})}$ is the effect of temperature on the maintenance respiration of roots in soil layer j, $C_{\mathrm{stem,pod(td)}}$ is the effect of temperature on the maintenance respiration of stems and pods, $\mathrm{BIO}_{\mathrm{root,\,j\,(td)}}$ is the biomass of the roots (kg ha^{-1}) in soil layer j, $\mathrm{BIO}_{\mathrm{stem,pod(td)}}$ is the biomass of stems and pods (kg ha^{-1}) on that day, and nsol is the number of soil layers (2 in this study). $\mathrm{C}_{\mathrm{root},j(\mathrm{td})}$ and $\mathrm{C}_{\mathrm{stem,pod(td)}}$ are given as

$$C_{\mathrm{root},j(\mathrm{td})} = 2.0^{\left(\frac{T_{\mathrm{sol},j(\mathrm{td})}-25}{10}\right)}\left\{1+\exp\left[s_3\left(T_{\mathrm{sol},j(\mathrm{td})}-s_4\right)\right]\right\}, \quad (37)$$

$$C_{\mathrm{stem(td)}} = 2.0^{\left(\frac{T_{a(\mathrm{td})}-25}{10}\right)}\left\{1+\exp\left[s_3\left(\mathrm{TM}_{\mathrm{td}}-s_4\right)\right]\right\}, \quad (38)$$

where $T_{\mathrm{sol},j\,(\mathrm{td})}$ is the temperature (°C) of soil layer j on a given day td, $\mathrm{TM}_{\mathrm{td}}$ is the average air temperature (°C) on td, and s_3 and s_4 are parameters that describe the shape of the curve. $T_{\mathrm{sol},j\,(\mathrm{td})}$ is calculated using the SWAT subroutine[7].

The growth respiration is calculated as

$$\mathrm{RG}_{\mathrm{td}} = \Delta\mathrm{Bio}_{\mathrm{td}} \times \mathrm{rg}, \quad (39)$$

where $\mathrm{RG}_{\mathrm{td}}$ is the growth respiration (kg ha^{-1} day^{-1}) on a given day *td*, $\Delta\mathrm{Bio}_{\mathrm{td}}$ is the actual biomass increment per day (kg ha^{-1} day^{-1}), and rg is the growth respiration rate (kg kg^{-1} day^{-1}).

3.3. Soil water balance and water stress

The soil water balance was modeled using a method similar to that described by Neitsch *et al.* (2005)[7] with two soil layers and no lateral flow. In this method, the water content in each soil layer is updated daily to account for rainfall, snowmelt, sublimation, transpiration, evaporation, and percolation. However, because soybean is a leguminous plant, our model did not consider the nitrogen cycle. The irrigation process was also not considered. Because we integrated the crop model and the SWAT model into our model, which could result in inconsistencies (especially with regard to the effects of water stress on the uncorrected photosynthetic rate), we introduced the following parameter to correct for the water stress factor. The distribution of this parameter was estimated using the MCMC procedure as follows:

$$W_{\mathrm{stress(td)}} = \min\left(w_{\mathrm{coef}} \times \mathrm{WSTRS}, 1.0\right), \quad (40)$$

where $W_{\mathrm{stress(td)}}$ is water stress, which is multiplied by v_{cmax} and j_{max} in equation 32 and 33; WSTRS is the water stress factor (calculated by the SWAT subroutine), ranging from 0 (high water stress; crops cannot grow) to 1 (no water stress) for each day; and w_{coef} is the water stress compensation coefficient estimated using the MCMC procedure.

3.4. Correction of parameters according to CO_2 concentration

The correction of parameters based on CO_2 concentration is easily included in the model using the following equation:

$$\mathrm{bio}_{\mathrm{coef}(C\mathrm{a})} = \mathrm{bio}_{\mathrm{coef}}\left[1 + \mathrm{dr}\left(\frac{C_{\mathrm{a}}}{c_{\mathrm{base}}} - 1\right)\right], \quad (41)$$

where $\mathrm{bio}_{\mathrm{coef}}$ is the biomass growth compensation coefficient and the variable on the left side of the equation ($\mathrm{bio}_{\mathrm{coef}(C\mathrm{a})}$) represents the corrected value of the corresponding parameter on the right side of the equation. The variable C_{a} represents atmospheric CO_2 (mol mol^{-1}), c_{base} is the baseline atmospheric CO_2 (mol mol^{-1}), and dr is parameters that describe the slopes of the linear function. In this model, $\mathrm{bio}_{\mathrm{coef}(C\mathrm{a})}$ decreases linearly with increasing atmospheric CO_2. If dr goes to zero, $\mathrm{bio}_{\mathrm{coef}(C\mathrm{a})}$does not change with atmospheric CO_2 and equal $\mathrm{bio}_{\mathrm{coef}}$. If $\mathrm{bio}_{\mathrm{coef}(C\mathrm{a})}$ decreases with increasing atmospheric CO_2, then dr will be negative (and vice versa). For example, if the value of dr is –0.2, the value of $\mathrm{bio}_{\mathrm{coef}(C\mathrm{a})}$ will decrease by 20% when atmospheric CO_2 is $2c_{\mathrm{base}}$ compared to the value of $\mathrm{bio}_{\mathrm{coef}(C\mathrm{a})}$ when atmospheric CO_2 is c_{base}.

4. Yield trends

When using historical yield data to calibrate the model parameters, we must consider temporal trends in the effects of nonclimatic factors. Crop yield should improve from year to year as a result of agricultural factors, such as changes in harvest loss, crop cultivar, and pesticide use. We therefore assumed the following linear trend in nonclimatic effects when evaluating the long-term yield data:

$$\text{Yield}_{\text{year}}=\text{base}_{\text{coef}}\cdot\text{Yield}\cdot\left[1+\text{nonc}_{\text{year}}(\text{Year-year}_{\text{base}})\right], \quad (42)$$

where $\text{Yield}_{\text{year}}$ is the estimated yield for a given year; $\text{base}_{\text{coef}}$ is the yield compensation coefficient, which must be calibrated for each spatial grid cell; Yield is the yield that is calculated by above models; $\text{nonc}_{\text{year}}$ is the annual rate of increase in the yield trend due to nonclimatic factors (which also must be calibrated for each grid); Year is the year; and $\text{year}_{\text{base}}$ is the criterion year (1980).

5. Cold stress

Applying the model to regional-scale yield prediction requires accounting for the effect of cold stress on crop yields particularly in high-latitude region. We accounted for cold stress using the following simple equation:

$$\text{Yield}'_{\text{year}} = \text{Yield}_{\text{year}}+\text{cold}_{\text{coef1}}\cdot\text{TM}_{\text{cold}}+\text{cold}_{\text{coef2}} \ , \quad (43)$$

where $\text{Yield}'_{\text{year}}$ is the estimated yield for the year in which cold stress is being considered; $\text{Yield}_{\text{year}}$ is the estimated yield for that year before correcting for cold stress; $\text{cold}_{\text{coef1}}$ and $\text{cold}_{\text{coef2}}$ are the slope and intercept, respectively, of the linear model of cold stress effect on the crop yield; and TM_{cold} is the weighted average of the temperatures during the growing season. The parameter TM_{cold} is weighted according to the degree of development of the crop (Fthu_{td}), which has a normal distribution with a mean of cold_{av} and a variance of cold_{va}.

6. Structure of the error term for MCMC using FACE data

For simplicity, we assumed a multivariate normal distribution as the error distribution for aboveground biomass and leaf biomass in our parameter estimation. We set the nondiagonal elements of the variance–covariance matrix of the error distribution to zero; that is, we assumed no correlation among the errors (see below for details). The values of the diagonal elements of the variance–covariance matrix were estimated on the assumption that the values were equivalent for each type of data (aboveground biomass and leaf biomass); *i.e.*, we estimated the values of two parameters associated with the error distribution. These simplifications allowed us to reduce the number of parameters to be estimated and to compute the parameter estimations in a reasonable amount of time. However, for the observed biomass error, it was not reasonable to assume that the variance of the error distribution was equal throughout the growing period. For example, the observation error distributions may differ between a biomass of 500 kg ha^{-1} and a biomass of 5000 kg ha^{-1}. Therefore, we used the following equations to calculate the variance of the biomass error distributions:

$$\sigma_{1,i} = \sigma_{\text{Bio(leef)}}\cdot\text{Bio}_{\text{leaf},i} \quad (44)$$

$$\sigma_{2,i} = \sigma_{\text{Bio(above)}}\cdot\text{Bio}_{\text{above},i}\ , \quad (45)$$

where $\sigma_{1,i}$ and $\sigma_{2,i}$ are the variances of the error distributions for leaf biomass and aboveground biomass, respectively, on day *i*; $\text{Bio}_{\text{leaf},i}$ and $\text{Bio}_{\text{above},i}$ are leaf biomass and aboveground biomass,

respectively; and $\sigma_{Bio(leaf)}$ and $\sigma_{Bio(above)}$ are the correction parameters for the variances of the error distributions for leaf biomass and aboveground biomass, respectively.

Additionally, we adopted a hierarchical structure in our statistical model. Because we analyzed time-series data from three years, we believe it is reasonable to assume that there were correlations among the errors in a given year. For example, the factors that were not considered in the model (such as pest damage) may have had different effects in different years. Thus, we adopted a hierarchical model in which the years (2001, 2002, and 2003) were included as random effects that were multiplied by the estimated biomass. For these random effects, we assumed normal distributions and estimated the variance of each distribution. The estimated parameters are listed in Supplementary Tables S1 together with the constant parameters. The estimated values of the parameters are listed in Supplementary Table S2.

7. Structure of the error term for MCMC using county level-data

For simplicity, we assumed a multivariate normal distribution as the error distribution for yield in our parameter estimation as above for each grid. We estimated the values of the parameter associated with the variance of error distribution for each grid. The estimated parameters are listed in Supplementary Tables S3.

8. Relationship between leaf ratio and heat unit rate

In this model, the leaf biomass at a given day is determined according to the relationship between $Rate_{leaf}$ (the ratio of leaf biomass to aboveground biomass) and $Fthu_{td}$ (the ratio of the total number of heat units 'to date' to the total number of heat units needed for maturity) on a given day, as described above (Eq. 8). Although such an allometric type of structure determination reduces the number of parameters in the model, the robustness of the parameter is important for estimating the crop biomass because the leaf biomass directly affects the absorbed PAR and thus the daily growth of the crop.

To confirm the robustness of the value of *l*, we also analyzed the data for the aboveground biomass and leaf biomass of soybean at multiple sites, over multiple years, of multiple cultivars[18]. These data were observed at four sites in Japan from 1978 to 1982 for six cultivars (two cultivars at each site). General information about each site and the cultivars used is presented in Table S4. These observations were carried out in each Prefectural Agricultural Experimental Station as part of a project called the "Green Energy Program" by the Ministry of Agriculture, Forestry and Fisheries of Japan. For each site, year, and cultivar, the biomasses of the leaf, root, stem, and pod were measured every 2 or 3 weeks during the growing season. The values of $Rate_{leaf}$ were calculated by dividing the observed leaf biomass by the aboveground biomass at each observation time. The values of $Fthu_{td}$ were calculated by assuming that $Fthu_{td}$ = 1 when leaf biomass = 0.

Figure S1 presents the relationship between $Rate_{leaf}$ and $Fthu_{td}$, in which the sites and years are not distinguished. The value of l estimated from this relationship was 0.77. Figure S2 presents the relationship between $Rate_{leaf}$ and $Fthu_{td}$ using the data of Morgan *et al.*[19]. This relationship appears to be robust, even if the atmospheric CO_2 concentration was increased. The estimated value of *l* from this relationship is 0.78. These results show the robustness of the relationship between $Rate_{leaf}$ and $Fthu_{td}$ in various cultivars and environmental conditions.

Supplementary Table S1. Estimated and fixed parameters. This list includes the parameters that were estimated with the MCMC method using biomass data from the FACE experiment. The averages and standard deviations of the prior distributions of the parameters are also provided. To simplify the model, we used normal distributions as the prior probability distributions for the estimated parameters.

Parameter	Definition	Estimated or fixed	Fixed value	Average	SD	Source
m	Parameter in the Ball–Berry model	Estimated	-	1.075×10^{1}	5.023×10^{1}	[20]
adj_E	Adjustment parameter for temperature effect	Estimated	-	1.000	7.000×10^{2}	Referred to as the standard deviation of the rate of change of E_{vcmax}[16]
bio_{init}	Initial biomass	Estimated	-	non-info	-	-
w_{coef}	Water stress compensation coefficient	Estimated	-	non-info	-	-
bio_{coef}	Biomass growth compensation coefficient	Estimated	-	non-info	-	-
dr	Correction factor for bio_{coef} at elevated atmospheric CO_2 concentrations	Estimated	-	non-info	-	-
thu_{total}	Total number of heat units needed for maturity	Estimated	-	non-info	-	Calculated using the daily average temperature and the value of tm_{base} (8 °C) for each year
q	Shape parameter in Eq. 2.5.3	Fixed	2.000	-	-	[8]
v_{cmax}	Maximum carboxylation rate	Fixed	9.776×10^{1}	-	-	[16]
j_{max}	Potential rate of electron transport	Fixed	2.179×10^{2}	-	-	[16]
E_{vcmax}	Shape parameter for the Arrhenius model	Fixed	6.533×10^{4}	-	-	[21]
E_{jmax}	Shape parameter for peaked model	Fixed	3.700×10^{4}	-	-	[21]
S_{jmax}	Shape parameter for peaked model	Fixed	6.500×10^{2}	-	-	[21]
H_{jmax}	Shape parameter for peaked model	Fixed	2.000×10^{5}	-	-	[21]
α	Quantum yield of electron transport in C_3 plants	Fixed	3.50×10^{-1}	-	-	Considering a photosynthetic quantum of 0.093 and an average canopy reflectance of 7%[16, 22]
l	Shape parameter for curve in the leaf-ratio function	Fixed	0.780	-	-	[19]
cf	Conversion factor for solar radiation at 550 nm (green)	Fixed	4.600×10^{6}			[22]
b'	Parameter in the Ball–Berry model	Fixed	1.962×10^{-2}	-	-	[20]

c_{base}	Baseline atmospheric CO_2 concentration	Fixed	3.733×10^{2}	-	-	[19]
k	Light extinction coefficient	Fixed	6.500×10^{-1}	-	-	[7]
l_{emerge}	Time of emergence	Fixed	7.170	-	-	[10]
nsol	Total number of soil layers	Fixed	2	-	-	-
tm_{base}	Baseline crop temperature	Fixed	8.000	-	-	[23]
rd	Leaf respiration factor	Fixed	1.500×10^{-2}	-	-	[8]
rm	Maintenance respiration rate	Fixed	9.164×10^{-3}	-	-	[24]
rg	Growth respiration rate	Fixed	5.706×10^{-2}	-	-	[24]
s_3	Shape parameter in Eq. 2.5.3	Fixed	1.300	-	-	[8]
s_4	Shape parameter in Eq. 2.5.3	Fixed	5.500×10^{1}	-	-	[8]
sla	Specific leaf area (ha kg^{-1})	Fixed	2.946×10^{-3}	-	-	[25]
hi	Harvest index	Fixed	3.900×10^{-1}	-	-	[19]
$coef_{sla}$	Adjustment parameter of sla for high CO_2 concentrations	Fixed	-2.800×10^{-1}	-	-	[11]
$coef_{hi}$	Adjustment parameter of hi for high CO_2 concentrations	Fixed	-2.900×10^{-1}	-	-	[11]
$base_{coef}$	Yield compensation coefficient	Fixed	1.000	-	-	-
$nonc_{year}$	Annual increase rate of the yield trend of nonclimatic factors	Fixed	0.000	-	-	-
$year_{base}$	Criterion year	Fixed	1980	-	-	-
$cold_{coef1}$	Shape parameter of cold stress function	Fixed	2.504×10^{1}	-	-	[26]
$cold_{coef2}$	Shape parameter of cold stress function	Fixed	4.897×10^{2}	-	-	[26]
$cold_{av}$	Average of weighting function	Fixed	5.000×10^{-1}	-	-	[b]
$cold_{va}$	Variance of weighting function	Fixed	1.000×10^{-1}	-	-	[b]

[a] Non-informative prior distribution.

[b] We did not account for cold stress when estimating the model parameters using the FACE data because there were no years in which the average temperature during the growing season was sufficiently low to significantly affect crop yields[26]. We did, however, account for cold stress when calibrating the model parameters using the historical yield data, because we included the grid cells for which the average temperatures were low.

Supplementary Table S2. Values of the model parameters estimated with the MCMC method using data from the FACE experiment. The values were estimated using the model selected in the model selection analysis.

Parameter[a]	Mean	SD
dr (correction for bio_{coef})	–0.20	0.05
bio_{init}	7.42	3.30
adj_E	1.00	0.06
m	10.77	0.47
bio_{coef}	0.57	0.03
w_{coef}	6.11	2.29

[a] The scale parameters of the error distribution (three parameters) and the hyper-parameter of the hierarchical model are not listed.

Supplementary Table S3. Parameters that were updated by the MCMC model using historical soybean yield data for each grid cell.

**Parameter**[a]	**Explanation**
dr	Correction factor for bio_{coef} at elevated atmospheric CO_2 concentrations
m	Parameter in the Ball–Berry model
w_{coef}	Water stress compensation coefficient
$nonc_{year}$	Annual rate of increase in yield trend due to nonclimatic factor(s)
$base_{coef}$	Yield compensation coefficient
$cold_{av}$	Average of weighting function
$cold_{va}$	Variance of weighting function

[a] The scale parameters of the error distribution (three parameters) and the hyper-parameter of the hierarchical model are not listed.

Supplementary Table S4. Site information used for further analysis of the robustness of the value of *l*.

Site	Latitude	Longitude	Elevation	Cultivar
Sapporo	43°00′	141°25′	70 m asl	Kitamusume, Wasesiroge
Daisen	39°32′	140°22′	30 m asl	Enrei, Wasesiroge
Shiojiri	36°07′	137°57′	710 m asl	Enrei, Nakasennari
Kumamoto	32°53′	130°45′	80 m asl	Akiyoshi, Enrei

Supplementary Figure S1. Relationship between leaf ratio and Fthu. This plot was developed using data from the Green Energy Program (AFFRCS, 1987). The dashed line indicates $l = 0.77$. The years and sites of observations are not distinguished in this plot.

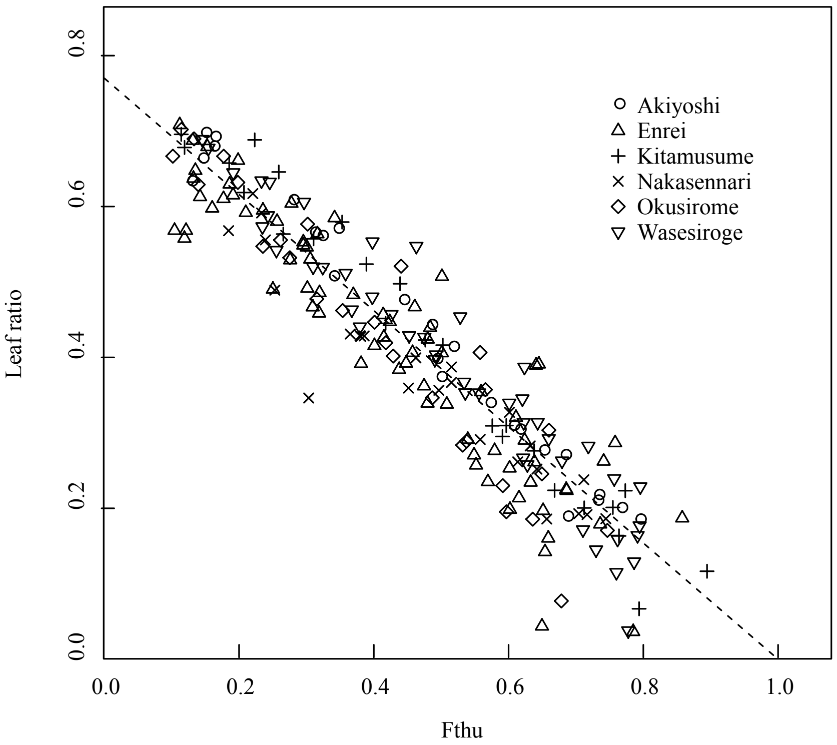

Supplementary Figure S2. Relationship between leaf ratio (the ratio of leaf biomass to aboveground biomass) and Fthu. This plot was developed using the data of Morgan *et al*. (2005). The dashed line indicates $l = 0.78$.

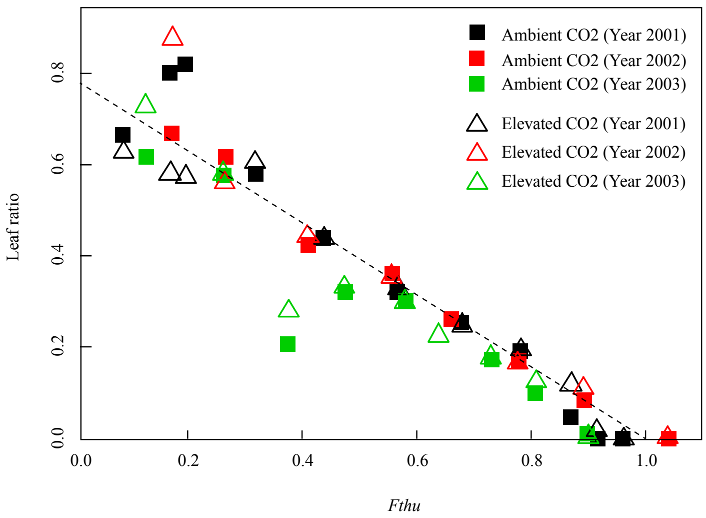

References

1. Iizumi, T., Yokozawa, M. & Nishimori, M., Parameter estimation and uncertainty analysis of a large-scale crop model for paddy rice: Application of a Bayesian approach. *Agric. For. Meteorol.* **149**, 333–348 (2009).
2. Farquhar, G. D., von Caemmerer, S. & Berry, J. A., A biochemical model of photosynthetic CO_2 assimilation in leaves of c3 species. *Planta* **149**, 78–90 (1980).
3. Baldocchi, D., An analytical solution for coupled leaf photosynthesis and stomatal conductance models. *Tree Physiol.* **14**, 1069–1079 (1994).
4. Collatz, G. J., Ball, J. T., Grivet, C. & Berry, J. A., Regulation of stomatal conductance and transpiration: a physiological model of canopy processes. *Agric. For. Meteorol.* **54**, 107–136 (1991).
5. Lokupitiya, E. *et al.* Incorporation of crop phenology in simple biosphere model (sibcrop) to improve land-atmosphere carbon exchanges from croplands. *Biogeosciences* **6**, 969–986 (2009).
6. Kosugi, Y., Shibata, S. & Kobashi, S., Parameterization of the CO_2 and H_2O gas exchange of several temperate deciduous broadleaved trees at the leaf scale considering seasonal changes. *Plant Cell Environ.* **26**, 285–301 (2003).
7. Neitsch, S. L., Arnold, J. G., Kiniry, J. R., Williams, J. R. & King, K. W. *Soil and water assessment tool theoretical documentation (version 2005)*. (United States Department of Agriculture, 2005).
8. Sellers, P. J. *et al.,* A revised land surface parameterization (SiB2) for atmospheric GCMs. Part I: Model Formulation. *J. Climate,* **9**, 676–705 (1996a).
9. Sellers, P. J. *et al.,* A revised land surface parameterization (SiB2) for atmospheric GCMs. Part II: The generation of global fields of terrestrial biophysical parameters from satellite data. *J. of Climate,* **9**, 706–737 (1996b).
10. Arif, M., Jan, M. T., Marwat, K. B. & Azim Khan, M. M., Seed priming improves emergence and yield of soybean. *Pakistan J. Bot.* **40**, 1169–1177 (2008).
11. Ainsworth, E. A. *et al*., A meta-analysis of elevated [CO_2] effects on soybean (*Glycine max*) physiology, growth and yield. *Glob. Change Biol.* **8**, 695–709 (2002).
12. Jones, C. A., *C-4 Grasses and Cereals*. John Wiley & Sons Inc. (1985).
13. Goudriaan, J. & Van Laar, H. H., *Modelling potential crop growth processes*. Kluwer Academic Publishers (1994).
14. Matthews, R. B. & Hunt, L. A., A model describing the growth of cassava (*Manihot esculenta L Crantz*). *Field Crop. Res.* **36**, 69–84 (1994).
15. Monsi, M. & Saeki, T. Über den Lichtfaktor in den Pflanzengesellschaften und seine Bedeutung für die Stoffproduktion. *J. Jpn. Bot.,* **14**, 22–52 (1953).
16. Medlyn, B. E. *et al*., Temperature response of parameters of a biochemically based model of photosynthesis. II. A review of experimental data. *Plant Cell Environ.* **25**, 1167–1179 (2002).
17. Ball, J. T., *An analysis of stomatal conductance.* Unpublished PhD thesis, Stanford University (1988).
18. Agriculture, Forestry and Fisheries Research Council Secretariat, Efficient use of solar energy in plant population of C3, C4 and CAM plant groups. Bulletin of Green Energy Program Group -II (Fixation of Matter) No. 13 (1987).
19. Morgan, P. B., Bollero, G. A., Nelson, R. L., Dohleman, F. G. & Long, S. P., Smaller than predicted increase in aboveground net primary production and yield of field-grown

soybean under fully open-air [CO_2] elevation. *Glob. Change Biol.* **11**, 1856–1865 (2005).
20. Leakey, A. D., Bernacchi, C. J., Ort, D. R. & Long, S. P., Long-term growth of soybean at elevated [CO2] does not cause acclimation of stomatal conductance under fully open-air conditions. *Plant Cell Environ.,* **29**, 1794–1800 (2006).
21. Kaschuk, G., Yin, X., Hungria, M., Leffelaar, P. A., Giller, K. E. & Kuyper, T. W., Photosynthetic adaptation of soybean due to varying effectiveness of N2 fixation by two distinct *Bradyrhizobium japonicum* strains. *Environ. Exp. Bot.*, 76, 1–6 (2012).
22. Dermody, O., Long, S. P., Kelly, L., Ughay, M. & DeLucia, E. H., How do elevated CO_2 and O_3 affect the interception and utilization of radiation by a soybean canopy? *Glob. Change Biol.* **14**, 556–564 (2008).
23. Yin, X. & van Laar, H. H. *Crop Systems Dynamics: An Ecophysiological Simulation Model for Genotype-by-Environment*. Wageningen Academic Publishers, Wageningen, The Netherlands (2005).
24. Bunce, J.A. & Ziska, L. H. Responses of respiration to increases in carbon dioxide concentration and temperature in three soybean cultivars. *Annal. Bot.,* **77**, 507–514 (1996).
25. Pedersen, P. & Lauer, J. G. Soybean growth and development in various management systems and planting dates. *Crop Sci.,* **44**, 508–515 (2004).
26. Horiguchi, I. The damage forecast of rice and soybean yield caused by cool summer temperature. *Hokkaido Daigaku Nogakubu Hobun Kiyo / Memoirs of the Faculty of Agriculture,* Hokkaido University*,* **12**, 222–229 (1981).
